# Glucocerebrosidase Mutations Cause Mitochondrial and Lysosomal Dysfunction in Parkinson’s Disease: Pathogenesis and Therapeutic Implications

**DOI:** 10.3389/fnagi.2022.851135

**Published:** 2022-03-23

**Authors:** Wei Zheng, Dongsheng Fan

**Affiliations:** ^1^Department of Neurology, Peking University Third Hospital, Beijing, China; ^2^Beijing Key Laboratory of Biomarker and Translational Research in Neurodegenerative Diseases, Beijing, China; ^3^Key Laboratory for Neuroscience, National Health Commission/Ministry of Education, Peking University, Beijing, China

**Keywords:** glucocerebrosidase (*GBA*), mitochondrial, lysosomal, Parkinson’s disease (PD), Gaucher’s disease (GD)

## Abstract

Parkinson’s disease (PD) is the second most common neurodegenerative disease and is characterized by multiple motor and non-motor symptoms. Mutations in the glucocerebrosidase (*GBA*) gene, which encodes the lysosomal enzyme glucocerebrosidase (GCase), which hydrolyzes glucosylceramide (GlcCer) to glucose and ceramide, are the most important and common genetic PD risk factors discovered to date. Homozygous *GBA* mutations result in the most common lysosomal storage disorder, Gaucher’s disease (GD), which is classified according to the presence (neuronopathic types, type 2 and 3 GD) or absence (non-neuronopathic type, type 1 GD) of neurological symptoms. The clinical manifestations of PD in patients with *GBA* mutations are indistinguishable from those of sporadic PD at the individual level. However, accumulating data have indicated that *GBA*-associated PD patients exhibit a younger age of onset and a greater risk for cognitive impairment and psychiatric symptoms. The mechanisms underlying the increased risk of developing PD in *GBA* mutant carriers are currently unclear. Contributors to *GBA*-PD pathogenesis may include mitochondrial dysfunction, autophagy-lysosomal dysfunction, altered lipid homeostasis and enhanced α-synuclein aggregation. Therapeutic strategies for PD and GD targeting mutant GCase mainly include enzyme replacement, substrate reduction, gene and pharmacological small-molecule chaperones. Emerging clinical, genetic and pathogenic studies on *GBA* mutations and PD are making significant contributions to our understanding of PD-associated pathogenetic pathways, and further elucidating the interactions between GCase activity and neurodegeneration may improve therapeutic approaches for slowing PD progression.

## Introduction

Parkinson’s disease (PD) is a heterogeneous neurodegenerative disorder characterized by the motor manifestations of resting tremor, rigidity, bradykinesia and postural instability, as well as some non-motor symptoms. Degeneration of dopaminergic neurons in the substantia nigra pars compacta and the presence of α-synuclein aggregates, that is, Lewy bodies, are the two main pathological hallmarks of PD. Many etiological factors, including multiple genetic causes and environmental and aging factors, have been demonstrated to underlie dopaminergic neurodegeneration (Obeso et al., 2017). More than 20 genetic loci that contribute to familial PD, which accounts for 5–10% of PD cases, have been discovered to date (Balestrino and Schapira, 2018). Glucocerebrosidase (*GBA*) gene mutations are currently recognized as the most important and common risk factors for developing PD (Sidransky and Lopez, 2012). The *GBA* gene mainly encodes the lysosomal enzyme glucocerebrosidase (GCase), which hydrolyzes glucosylceramide (GlcCer) to glucose and ceramide. In addition, GCase catalyzes the transfer of glucose from GlcCer to cholesterol to contribute to in the synthesis of β-cholesteryl glucoside (Franco et al., 2018). The *GBA* gene is located on chromosome 1q21 and is composed of 11 exons and 10 introns spanning a 7.6 kb sequence (Gan-Or et al., 2018). More than 300 known *GBA* mutations, among which L444P and N370S are the most common, have been found (O’Regan et al., 2017). The N370S mutation is most common in the Ashkenazi Jewish population and is often regarded as a mild mutation (Sidransky and Lopez, 2012), whereas the L444P mutation is found worldwide and is often regarded as a severe mutation (Gan-Or et al., 2008; Do et al., 2019). Homozygous mutations in *GBA* result in the most common lysosomal storage disorder, Gaucher’s disease (GD) (Grabowski, 2008). Approximately, 7–12% of PD patients carry *GBA* mutations (García-Sanz et al., 2021), and 25% of GD patients have a first- or second-degree relative with PD (Aflaki et al., 2017). A large multicenter study estimated that the odds ratio of PD patients harboring a *GBA* mutation was 5.43 compared with that of controls, confirming that *GBA* mutation is the single largest risk factor for developing PD identified to date (Sidransky et al., 2009). The proportion of *GBA* mutation carriers among PD patients ranges depending on the population studied and whether the whole exome is sequenced, with highest frequencies found among the Ashkenazi Jewish population (Schapira et al., 2016).

To date, the mechanisms that underlie *GBA* mutations that increase the risk of developing PD have not been fully elucidated. Several perspectives have been provided, including enhanced SNCA aggregation, lysosomal dysfunction, impaired autophagy, altered lipid homeostasis and mitochondrial dysfunction. In this review, we discuss *GBA* mutations and PD and focus on the current advances in understanding the pathogenesis by which *GBA* mutations increase the risk for developing PD, with the goal of improving our understanding of the role of GCase deficiency in the neurodegeneration of PD and gaining further insights into the pathogenetic pathways in PD. Finally, we discuss the implications for PD therapy based on an investigation of the relationship between *GBA* and PD, with the goal of finding new therapeutic targets that slow PD progression.

## Gaucher’s Disease

Gaucher’s disease is a rare autosomal recessive disorder caused by insufficient activity of the lysosomal enzyme GCase (Stirnemann et al., 2017). A deficient GCase level leads to the accumulation of its substrate GlcCer in lysosomes of reticuloendothelial lineage, causing the acquisition of a variety of clinical phenotypes, including hepatosplenomegaly, anemia, thrombocytopenia, and bone disease (Grabowski et al., 2015). GD is a panethnic disease but is most common in the Ashkenazi Jewish population, which has an incidence of 1/800, significantly higher than the 1/40,000 to 1/60,000 incidence in the general population but rises to Grabowski (2008). GD is classified by the involvement neurological symptoms (neuronopathic types, type 2 and 3 GD) or not (non-neuronopathic type, type 1 GD) (Sidransky, 2004). Type 1 GD, the most common phenotype of GD, is considered to indicate the absence of neurological involvement. The symptoms of type 1 GD are variable and can include hepatosplenomegaly, fatigue, anemia, thrombocytopenia, pulmonary hypertension, and osteopenia/osteoporosis, and type 1 GD symptoms can appear at any age (Stirnemann et al., 2017). Type 2 or acute neuronopathic GD is the most severe form, often appearing in infancy, with organomegaly, pancytopenia, skin abnormalities and severe central nervous system (CNS) impairment, followed by death in the first years of life (Weiss et al., 2015). Type 3 or subacute neuronopathic GD primarily involves visceral signs and impaired horizontal saccadic eye movement and usually presents in adolescence (Stirnemann et al., 2017).

## *GBA* Mutations and Parkinson’s Disease

The association between *GBA* mutations and PD was first described in the 1990s, and GD patients also show concomitant parkinsonism (Neudorfer et al., 1996; Tayebi et al., 2003). A clinical study screened 99 Ashkenazi Jewish patients with idiopathic PD and 1543 healthy Ashkenazi Jewish individuals for six *GBA* mutations and found that 31.3% of the PD patients expressed one or two *GBA* mutant alleles, compared with 6.2% of the controls (Aharon-Peretz et al., 2004). Another study reported the genotyping of 57 subjects with PD using brain bank samples found that 12 samples (21%) obtained from PD patients showed alterations in the *GBA* gene (Lwin et al., 2004). Since these initial studies were reported, multiple studies with PD cohorts characterized by different regions and ethnic origins have been performed to determine the frequency of *GBA* mutations. The frequency of *GBA* mutations ranged from 10.7 to 31.3% in the Ashkenazi Jewish population with PD (Aharon-Peretz et al., 2004; Clark et al., 2005) but ranged from 2.3%, the lowest frequency and reported in the Norwegian population with PD, to 9.8% for individuals of other ethnic origins (Toft et al., 2006; Seto-Salvia et al., 2012). In fact, on the one hand, ethnic origin certainly contributes to different frequencies of *GBA* mutations in PD; on the other hand, the methods used for genotyping affect the identification of mutants, with some studies detecting specific common *GBA* mutations, for instance, N370S and L444P, and others sequencing all exons of the *GBA* gene.

Since most previous single-center PD cohort studies based on *GBA* mutations were limited in the number of ethnic populations included, *GBA* genotyping methods and sample size, an international multicenter collaborative study including 5691 PD patients (780 were Ashkenazi Jewish patients) and 4898 controls (387 were Ashkenazi Jewish patients) from 16 centers was performed in 2009 (Sidransky et al., 2009). All the centers screened at least two mutations, N370S and L444P, and found that 15% of the PD patients carried *GBA* mutations compared with 3% of the controls among the Ashkenazi Jewish patients; 3% of the PD patients carried *GBA* mutations compared with 1% of the controls among non-Ashkenazi patients (Sidransky et al., 2009). Importantly, when *GBA* was fully screened in 1883 non-Ashkenazi Jewish patients and 1611 non-Ashkenazi Jewish controls, 7% of the patients were found to have *GBA* mutations, indicating that approximately 45% of the mutant loci could be missed when only N370S and L444P are sequenced (Sidransky et al., 2009). This study confirms that an adequate sample size and accurate genotyping are imperative to ascertain the frequency of *GBA* mutations among populations. In a large study with a European cohort, all exons of *GBA* were screened in 786 PD patients who had a familial PD history, 605 sporadic PD patients and 391 controls, and an overall *GBA* carrier frequency of 6.7% was found for the PD patients, including sporadic and familial subjects, compared with 1% of the control individuals (Lesage et al., 2011). Furthermore, *GBA* mutations were found more frequently in patients with a family history of PD (8.4%) than in isolated cases (5.3%) (Lesage et al., 2011).

## Clinical Features of *GBA*-Associated Parkinson’s Disease

The clinical manifestations of PD patients with *GBA* mutations are indistinguishable from those of sporadic PD patients on an individual level. However, when analyzing PD associated with *GBA* mutations in a group, patients with PD and *GBA* mutations exhibited a 1.7– 6-year earlier age of onset than those with idiopathic PD (Aharon-Peretz et al., 2004; Clark et al., 2007; Neumann et al., 2009; Nichols et al., 2009). PD patients with *GBA* mutations who had two mutant *GBA* alleles developed PD at an earlier age (54.2 versus 65.2 years) than patients who heterozygous, carrying one mutant allele (Alcalay et al., 2014). However, the age-specific risk of developing PD at age 60 and 80 years was higher in GD patients (4.4 and 9.1%) than in heterozygous individuals (1.5 and 7.7%) but this difference was not significant (Alcalay et al., 2014). These data suggested that a second *GBA* mutant allele contributes to a younger age of PD onset but does not increase the overall risk of developing PD. In addition, patients with PD and *GBA* mutations had a higher frequency of cognitive impairment or dementia (Neumann et al., 2009; Winder-Rhodes et al., 2013; Creese et al., 2018). Compared with idiopathic PD patients, *GBA* carriers with a severe mutation, such as L444P, presented with a fivefold greater risk for developing dementia, while those with a mild mutation, such as N370S, exhibited a twofold greater risk for developing dementia (Cilia et al., 2016), suggesting that the extent of cognitive impairment differs on the basis of the severity of mutations. PD patients with *GBA* mutations were also found to be susceptible to psychiatric symptoms, including depression, hallucinations, sleep disturbances and anxiety (Brockmann et al., 2011). However, the clinical features of *GBA*-associated PD resemble those of idiopathic PD in terms of tremor, rigidity, and bradykinesia (Clark et al., 2007; Westbroek et al., 2011). In other studies, PD patients carrying *GBA* mutations were reported to be less likely to have tremor at onset but more likely to present bradykinesia at onset (Gan-Or et al., 2010; Lesage et al., 2011; Swan and Saunders-Pullman, 2013), which may suggest the need to expand the sample size and extend the follow-up times. In addition to an earlier age of onset, more severe symptoms and more rapid progression have been observed in PD patients carrying *GBA* mutations than in idiopathic PD patients in most studies (Mata et al., 2016).

## Proposed Pathogenesis of *GBA*-Associated Parkinson’s Disease

Recent studies have been exploring the contribution of mutant *GBA* to PD pathogenesis. To date, the mechanisms that underlie the increased risk of developing PD among *GBA* mutation carriers have not been fully elucidated. In general, in autosomal dominant forms of PD, such as those involving LRRK2 and α-synuclein, gain-of-function mutations are usually involved in PD pathogenesis. In contrast, loss-of-function mutations, such as those in such as parkin, DJ-1 and PINK1, are implicated in most autosomal recessive forms of PD.

**FIGURE 1:**
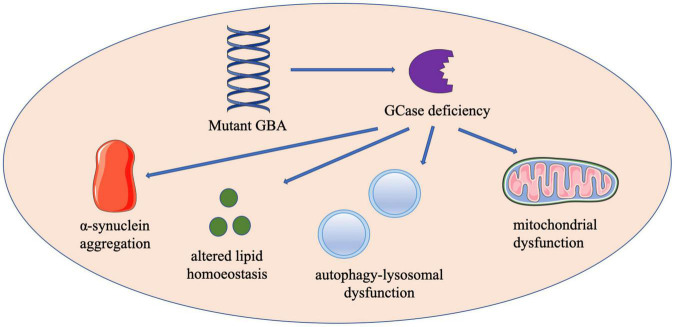
The schematic diagram of proposed mechanisms by which *GBA* mutations contribute to the development of PD. Contributions to *GBA*-PD pathogenesis may include enhanced α-synuclein aggregation, altered lipid homeostasis, autophagy-lysosomal dysfunction and mitochondrial dysfunction. *GBA*, glucocerebrosidase; GCase, glucocerebrosidase.

Notably, the inheritance of *GBA*-associated PD does not follow strict Mendelian law, although GD is an autosomal recessive disease. Therefore, both gain-of-function and loss-of-function have been proposed as explanations for *GBA* mutation increasing the risk of PD development (Figure 1).

## *GBA* Mutations and α-Synuclein Aggregation

Multiple studies point to the vital role of α-synuclein in the pathogenesis of *GBA*-associated PD, supporting a gain-of-function mechanism in which mutant GCase may directly lead to α-synuclein aggregation. In the brains of PD patients with *GBA* mutations, GCase is present in 32–90% (mean 75%) of Lewy bodies (Goker-Alpan et al., 2010). In contrast, GCase is positive in <10% of Lewy bodies of subjects without *GBA* mutations (Goker-Alpan et al., 2010), suggesting that mutant GCase may lead to the aberrant aggregation of α-synuclein, the major element in Lewy bodies. Several independent studies have detected α-synuclein accumulation in transgenic *GBA* mouse models (Migdalska-Richards et al., 2017b), inhibited GCase activity in conduritol-β-epoxide-treated SH-SY5Y cell cultures and mice (Manning-Bog et al., 2009; Cleeter et al., 2013), and induced pluripotent stem cell (iPSC)-derived neurons obtained from *GBA*-associated PD patients and GD patients (Schondorf et al., 2014; Woodard et al., 2014; Fernandes et al., 2016). Mazzulli et al. (2011) proposed that a reciprocal relationship between GCase and α-synuclein in synucleinopathies. GlcCer accumulation due to decreased activity of GCase may lead to aggregation of α-synuclein by stabilizing soluble toxic α-synuclein oligomeric intermediates, which tend to transition into insoluble deposits such as Lewy bodies; at the same time, elevation of α-synuclein levels inhibits lysosomal GCase maturation and activity, in turn augmenting α-synuclein aggregation, forming a self-propagating feedback loop in PD and other synucleinopathies that eventually leads to neurodegeneration (Mazzulli et al., 2011). Furthermore, in another study, by Kim et al. (2018) demonstrated that GCase deficiency due to *GBA* loss-of-function mutations influences the phase transition of α-synuclein. Accumulation of GlcCer resulting from *GBA* mutations increases α-synuclein monomers that tend to aggregate and convert into oligomers and destabilize α-synuclein tetramers and related multimers, which are resistant to aggregation (Kim et al., 2018). α-Synuclein, as the main pathological factor in PD, has been demonstrated to spread between neurons in a prion-like manner (Gomez-Benito et al., 2020). In addition to influencing the phase transition of α-synuclein, loss of *GBA* function was found to promote perpetual cell-to-cell transmission of α-synuclein aggregates, including both exogenous and endogenous α-synuclein aggregates (Bae et al., 2014).

Accumulation of α-synuclein not only inhibits lysosomal GCase maturation and activity but also affects the transport of GCase from the endoplasmic reticulum (ER) to lysosomes through the Golgi apparatus. In normal GCase-functioning cells, wild-type GCase is synthesized within polyribosomes on the ER and is translocated into the ER (Ron and Horowitz, 2005). Then, GCase is transported to the Golgi, from where to be transported to lysosomes (Ron and Horowitz, 2005). Lysosomes are the key organelles critical for the degradation of proteins, such as α-synuclein (Lawrence and Zoncu, 2019). In *GBA*-mutated cells, GlcCer accumulation due to lysosomal GCase deficiency causes lysosomal dysfunction, eventually leading to α-synuclein aggregation. The accumulation of α-synuclein can impair the transport of GCase, in turn reducing α-synuclein turnover (Schapira and Gegg, 2013). However, the mechanism by which α-synuclein affects GCase transport remains unclear. Lysosomal integral membrane protein type-2 (LIMP-2), the lysosomal integral membrane protein critical for transporting GCase from the ER to lysosomes, does not bind α-synuclein (Gegg et al., 2012). The level of LIMP-2 is unaffected in the substantia nigra of PD patients with *GBA* mutations (Gegg et al., 2012). These data indicate that we should consider whether the degree of GCase decrease depends on decreased transcription or increased degradation of mRNA and the role of α-synuclein in this process.

## *GBA* Mutations and Autophagy-Lysosomal Dysfunction

Lysosomes are acidic dynamic organelles involved in the degradation of harmful or unnecessary cellular contents, such as long-lived proteins and damaged organelles, *via* enzymatic degradation, and autophagic pathways, including macroautophagy, microautophagy and chaperone-mediated autophagy (Dehay et al., 2013; Chun and Kim, 2018). Disruption of the autophagy-lysosomal system has been identified in studies of PD pathogenesis in recent years. Given the lysosomal localization enzyme GCase, the link between GCase and autophagy-lysosomal pathways has attracted attention. Schondorf et al. (2014) found that iPSC-derived neurons from *GBA*-associated PD patients and GD patients exhibited autophagic defects due to impaired autophagosome-lysosome fusion. GCase influenced α-synuclein accumulation not only by enhancing the production of α-synuclein but also by decreasing the degradation of α-synuclein *via* autophagy-lysosomal dysfunction. Autophagy-lysosomal disruptions result in enlargement of the lysosomal compartment in iPSC-derived dopamine neurons of PD patients with *GBA*-N370S mutations, subsequently increasing the extracellular α-synuclein level (Fernandes et al., 2016). Consistent with iPSC-derived neurons with *GBA* mutations, impaired autophagy and dysfunctional lysosomes have been found in fibroblasts obtained from PD patients with *GBA* mutations (Garcia-Sanz et al., 2017). Additionally, *GBA* deficiency leads to α-synuclein accumulation by inhibiting autophagy in neuroblastoma cells (Du et al., 2015). Protein phosphatase 2A inactivation is involved in the suppression of autophagy induced by *GBA* mutants (Du et al., 2015). Importantly, the autophagy inducer rapamycin reversed α-synuclein accumulation in *GBA*-knockdown cells (Du et al., 2015). Defective autophagic clearance due to lysosomal dysfunction has also been detected in GD iPSC-derived neural cells (Awad et al., 2015). Further analysis revealed that decreased level of the transcription factor EB, the main regulator of lysosomal genes, impaired lysosomal biogenesis in GD iPSC-derived neural cells (Awad et al., 2015). Lysosomal function was rescued by recombinant GCase and the overexpressed transcription factor EB, but overexpression of the transcription factor EB alone did not restore lysosomal function, suggesting that GCase leads to autophagy-lysosomal dysfunction in part *via* transcription factor EB (Awad et al., 2015). Further studies are needed to investigate the role of autophagy-lysosomal dysfunction in *GBA*-related pathogenesis.

## *GBA* Mutations and Altered Lipid Homeostasis

Lipids, as the main component of the cell membrane, are central to maintaining membrane structure and cellular processes, including synaptic transmission and molecular trafficking. Accumulating evidence has demonstrated that lipid homeostasis and its interaction with GCase are involved in PD pathogenesis. GCase normally cleaves GlcCer into glucose and ceramide. Based on a loss-of-function mechanism, the accumulation of GlcCer substrates caused by a reduced GCase level promotes the formation of toxic α-synuclein aggregation by stabilizing α-synuclein oligomers, subsequently resulting in neurodegeneration (Mazzulli et al., 2011). Dysfunction of cholesterol metabolism could result in changes in lipid rafts, which play a vital role in synaptic function (García-Sanz et al., 2021). Utilizing iPSC-derived neuronal models, Zunke et al. (2018) revealed equilibrium between two α-synuclein forms, a 100 Å-sized high molecular weight conformer and a low-molecular-weight 35 Å-sized monomeric species. Glycosphingolipids preferentially convert high-molecular-weight α-synuclein conformers into compact, assembled toxic oligomers and do not convert high-molecular-weight species into monomers, as indicated by fewer interactions with low-molecular-weight monomeric species (Zunke et al., 2018). In addition, cholesterol accumulation was detected within the lysosomes of fibroblasts from patients with *GBA* mutant-associated PD (Garcia-Sanz et al., 2017). However, it is still unclear whether sphingolipids accumulate in other organelles and the exact location of sphingolipid accumulation. Mitochondria purified from *GBA*-knockout iPSC-derived neurons were demonstrated to have a significant accumulation of GlcCer and deacylated GlcCer glucosylsphingosine (Schondorf et al., 2018). Notably, only certain species of GlcCer were increased, with 65% increases in C16:0 and C24:0 species and a 30% reduction in C20:0 species (Fernandes et al., 2016). These data suggest that mutant GCase may promote the accumulation of certain GlcCer species and hydrolyze certain GlcCer species at the same time. However, the association between *GBA* mutations and lipid homeostasis remains controversial. Gegg et al. (2015) reported no changes in total GlcCer levels in the putamen or cerebellum in PD patients carrying *GBA* mutations compared with control individuals but observed loss of GCase activity. The putamen and cerebellum in PD patients with *GBA* mutations have previously been shown to exhibit decreased activity of GCase by 48 and 47%, respectively (Gegg et al., 2012). No GlcCer accumulation has been found in the brains of patients with sporadic PD, as GCase activity was decreased by 33% in the substantia nigra of patients with sporadic disease but decreased by 58% in the substantia nigra of *GBA*-PD patients (Gegg et al., 2012, 2015; Boutin et al., 2016). As lipidomic analyses were performed on postmortem brain tissue, the samples may have been contaminated with glia, which would discredit findings of GCase accumulation in neurons. Another explanation for the lack of lipid substrate accumulation upon loss of GCase activity is that the residual enzyme could prevent lipid accumulation. Further studies are needed to clarify whether GCase deficiency leads to GlcCer accumulation and whether functional compensatory pathways exist in mutant GCase cells.

## *GBA* Mutations and Mitochondrial Dysfunction

Mitochondrial dysfunction has long been thought to participate in PD pathogenesis. Accumulating evidence has shown that mutant GCase is associated with mitochondrial defects in GD and PD. In GD mouse models, mitochondria showed impaired function and morphology, exhibiting respiratory chain defects, a decreased mitochondrial membrane potential due to reversal of ATPase, and fragmented mitochondrial morphology (Osellame et al., 2013). Like mice with GD, N370S fibroblasts exhibit fragmented mitochondria and increased reactive oxygen production (Garcia-Sanz et al., 2017). GCase inhibition in a human dopaminergic cell line resulted in increased free radical formation and mitochondrial dysfunction, including reduced mitochondrial membrane potential and decreased adenosine diphosphate phosphorylation (Cleeter et al., 2013). Consistent with these findings, iPSC-derived neurons from *GBA*-associated PD patients showed reduced respiration, increased morphological changes, higher levels of reactive oxygen species and defects in mitochondrial dynamics (Schondorf et al., 2018). Interestingly, a gene dosage effect was not observed between heterozygous *GBA*-PD and *GBA*-knockout neurons, suggesting that different pathways may contribute to mitochondrial dysfunction in heterozygous and homozygous *GBA* carriers. One such pathway may be involved in the activation of the unfolded protein response and increased ER stress in *GBA*-PD neurons, as the complete loss of GCase in *GBA*-knockout cells was incapable of triggering ER stress, supporting gain-of-function mechanisms (Fernandes et al., 2016; Schondorf et al., 2018). Similarly, Yun et al. (2018) found reduced mitochondrial size, increased reactive oxygen species production and decreased complex I activity in heterozygous L444P *GBA*-knock-in mice. The L444P *GBA* heterozygous mutation increased the susceptibility of mice to loss of nigrostriatal dopaminergic neurons and mitochondrial damage following MPTP administration (Yun et al., 2018). Under normal conditions, damaged and fragmented mitochondria are degraded *via* mitophagy, a specific form of autophagy, with serine/threonine kinase PTEN-induced putative kinase protein 1 (PINK1) and the ubiquitination E3 ligase parkin playing pivotal roles (Youle and Narendra, 2011). When mitochondria are damaged, PINK1 accumulates on the outer membrane in response to decreased membrane potential, thus triggering recruitment of parkin for the elimination of damaged mitochondria (Narendra et al., 2008; Twig et al., 2008). However, mutant GCase has been observed to impair mitophagy in *GBA*-mutant cells, leading to the accumulation of damaged mitochondria and the production of reactive oxygen species, subsequently leading to neurodegeneration and, eventually, to cell death. Osellame et al. (2013) found defective mitophagy in *GBA*-knockout neurons and astrocytes because the mitochondrial membrane potential in the *GBA*-knockout cells was not low enough for parkin recruitment, which is recruited to damaged mitochondria when the mitochondrial membrane potential is dissipated. Furthermore, Li et al. (2019) reported that the L444P *GBA* mutation impaired mitophagy *via* a dual mechanism, impairing autophagy induction and abrogating damaged mitochondria priming, processes involved in triggering autophagy machinery recruitment to damaged mitochondria. The L444P *GBA* mutation was impeded both PINK1-parkin-dependent and PINK1-parkin-independent pathways (Li et al., 2019).

## Therapeutic Implications for *GBA*-Associated Parkinson’s Disease

### Enzyme-Replacement Therapy

Enzyme-replacement therapy (ERT) and substrate reduction therapy (SRT) are two Food and Drug Administration (FDA)-approved therapies for type I GD. ERT is now accepted as the first-line treatment of type I GD, and it is aimed at complementing deficient GCase enzyme activity upon intravenous administration (Shemesh et al., 2015). While ERT treatments significantly improve the visceral and hematologic symptoms of GD, current ERT treatments are ineffective against the neuropathic manifestations of GD since the enzyme does not cross the blood–brain barrier (BBB) (Boer et al., 2020). Thus, there are no data showing ERT treatments prevent the development of PD. To enhance ERT delivery to the CNS, Gramlich et al. (2016) tagged GCase with BBB-crossing peptides, and they effectively bound neurons, as expected. Another alternative is delivering therapeutic proteins through extracellular vesicles, such as exosomes, which are capable of transporting therapeutic proteins across the BBB (Hall et al., 2016). The system of delivering GCase across the BBB may increase the options for using ERT treatment to attenuate the neurological symptoms of GD or PD.

### Substrate Reduction Therapy

Substrate reduction therapy is accepted as the second-line treatment for GD with patients who are unsuitable for or unwilling to receive ERT; however, SRT leads to more side effects than ERT, the prominent of which are gastrointestinal and neurological symptoms such as onset of tremor or peripheral neuropathy (Cox et al., 2003; Hollak et al., 2009). SRT is administered to inhibit GlcCer synthase, reducing the level of lipid substrates, which effectively improves the hematological and visceral manifestations (Sardi et al., 2018). Miglustat and eliglustat are two SRT-approved drugs currently prescribed that can be administered orally (Shemesh et al., 2015; Mistry et al., 2017). Miglustat can partially cross the BBB and has been tested in an open-label randomized clinical trial for the treatment of GD type III; however, it did not significantly improve the neurological symptoms of GD type III (Schiffmann et al., 2008). SRT may be less effective than ERT in some aspects GD treatment; for example, hematological responses of miglustat are slower and of lesser magnitude compared to ERT, while the visceral symptom improvement obtained with miglustat is similar to that realized with ERT (Mistry et al., 2017). Furthermore, the novel GlcCer synthase inhibitor Genz-682452 has shown good CNS penetration and attenuation of several neuropathic and behavioral symptoms in GD mouse models (Marshall et al., 2016). Another brain-penetrating GlcCer synthase inhibitor, GZ667161, was found to reduce the levels of GlcCer and glucosylsphingosine in the CNS. Remarkably, prolonged administration of GZ667161 decreased α-synuclein accumulation and improved behavioral outcomes in synucleinopathy models (Sardi et al., 2017). Based on these studies, a multicenter, randomized, double-blind clinical study was launched to determine the safety and efficacy of the GlcCer synthase inhibitor GZ/SAR402671 in PD patients with *GBA* mutations (clinicalTrials.gov identifier: NCT02906020). These studies have provided promising therapeutic strategies for *GBA*-associated PD patients. However, SRT, which aims to reduce the levels of lipid substrates, does not prevent the other mechanisms that may play a role in the development of *GBA*-associated PD.

### Gene Therapy

In recent years, gene therapy, which aims to deliver corrected genetic material into human cells, has emerged as a novel therapeutic strategy for PD (Hitti et al., 2019). Adeno-associated virus (AAV) vector-mediated delivery of *GBA* (using AAV-*GBA*) has been reported to be effective in augmenting GCase expression and protecting neurons against α-synuclein-mediated neuronal damage in mice and macaques (Rocha et al., 2015; Sucunza et al., 2021). AAVs are popular due to their strong neuronal tropism and good safety profiles, as they are not known to integrate into the host genome (Hudry and Vandenberghe, 2019). Research reported by Sardi et al. (2013) showed that augmenting GCase activity with an AAV vector encoding human GCase in GD mice reduced the levels of lipid glucosylsphingosine and aggregated proteins, such as α-synuclein, tau, and ubiquitin. Hippocampal administration of AAV-mediated GCase reversed the memory deficit of GD mice (Sardi et al., 2013). Massaro et al. (2018) demonstrated that fetal intracranial injection of AAV-*GBA* into GD mice elevated the level of GCase and ameliorated neuroinflammation and neurodegeneration. AAV-*GBA* gene therapy also ameliorated motor coordination and microglial and astrocyte activation, which correlated with neuronal loss, in fetal mice and macaques (Massaro et al., 2018). However, GCase normalization did not completely abolish GD pathology; that is, it did not completely normalize brain glycosphingolipid levels or prevent long-term microglial and astrocyte activation (Massaro et al., 2018). Based on these findings, different transduction efficiencies of individual neurons and peripheral macrophages in a CNS pathology model should be taken into consideration. In addition, intravenous injection of AAV-PHP. B, encoding GBA, a novel engineered AAV-*GBA* vector, restored the level of GCase, prevented α-synuclein inclusion formation, and recovered the loss of lifespan and cognitive performance in A53T-SNCA mice (Morabito et al., 2017). Remarkably, AAV-PHP. B targeting the BBB did not alter BBB integrity or selectivity (Morabito et al., 2017). Altogether, AAV-*GBA* gene therapy enables non-invasive and effective expression of GCase, possessing great potential as a therapeutic for GD and PD.

However, the route of delivery, optimal serotype, different transduction efficiencies of individual neurons, accessibility to widespread neuronal circuits and potential side effects of long-term treatment with GCase need be investigated before AAV-*GBA* gene therapy is translated into the clinic.

## Pharmacological Small-Molecule Chaperones

Under normal conditions, GCase is subject to ER repair machinery that corrects folding or transported to lysosomes. When *GBA* is mutated, the number of misfolded proteins exceeds the ER capacity, which triggers the unfolded protein response and ER-associated degradation, through which misfolded enzymes are broken down, leading to a reduction in GCase protein resident in the lysosome (Meusser et al., 2005; Bendikov-Bar et al., 2011; Schapira and Gegg, 2013). Pharmacological small-molecule chaperones may selectively bind to mutant GCase and stabilize and correct misfolded GCase to facilitate protein tracking through the ER to lysosomes (Chen et al., 2020). Small-molecule chaperones also have the ability to prevent ER stress, subsequently attenuating apoptosis and mitochondrial dysfunction (de la Mata et al., 2015). Small-molecule chaperones can be divided into inhibitory chaperones, which interact with the active site of an enzyme, and non-inhibitory chaperones, which primarily enhance enzymatic activity.

Most small-molecule chaperones developed in recent studies are inhibitory chaperones that bind to the active site of misfolded GCase and facilitate its correct folding or translocation to lysosomes (Jung et al., 2016). However, when mutant GCase and its bound inhibitor reach lysosomes, the substrate GlcCer out-competes the inhibitor to optimize substrate degradation (Aflaki et al., 2017). This competition between inhibitors and substrates makes it a challenge to optimize drug dosing to balance the chaperoning and inhibitory capacity of inhibitory small-molecule chaperones for clinical applications. From this drug collection, ambroxol and isofagomine have shown their potential and have been investigated in preclinical and early-stage clinical studies. Ambroxol is a potential inhibitory chaperone candidate that has been selected *via* high-throughput screening of a library of FDA-approved drugs (Maegawa et al., 2009). Ambroxol, a cough medicine widely used to treat airway mucus hypersecretion and hyaline membrane disease in newborns, was identified as a mixed inhibitor of GCase activity. Ambroxol exerts its maximal activity in the ER where the pH is neutral and loses its binding affinity in lysosomes where the pH is acidic (Maegawa et al., 2009). This pH dependence of ambroxol prevents degradation blockades of the substrate, making ambroxol a desirable small molecule. Ambroxol has been demonstrated to increase GCase activity and lysosomal localization of mutant GCase significantly in various *in vitro* and *in vivo* models, such as patient cells (Bendikov-Bar et al., 2013; Luan et al., 2013; McNeill et al., 2014; Yang et al., 2017), mice (Sanders et al., 2013; Migdalska-Richards et al., 2016), and non-human primates (Migdalska-Richards et al., 2017a), in independent studies. In an open-label, non-randomized, non-controlled clinical trial with 17 PD patients, ambroxol crossed the BBB and bound to GCase and increased GCase protein levels and α-synuclein concentrations in the cerebrospinal fluid in patients both with and without *GBA* mutations, and it induced no serious adverse effects (Mullin et al., 2020). In addition, another clinical trial on ambroxol as a treatment for PD dementia is currently underway (clinicalTrials.gov identifier: NCT02914366). Another inhibitory chaperone is the iminosugar isofagomine, which binds and stabilizes GCase and increases cellular and lysosomal GCase levels (Lieberman et al., 2007). Treatment of patient cells and mice with isofagomine increased GCase activity in brain and visceral tissue, reduced GlcCer levels, attenuated proinflammatory responses, delayed the onset of neurological disease and extended the lifespan (Khanna et al., 2010; Sun et al., 2012). However, isofagomine did not lead to significant clinical improvement in 18 patients of a GD clinical study, and therefore, further development was discontinued, suggesting that more elaborate early-stage clinical trials are needed to ensure the safety and efficiency of isofagomine in GD or PD patients.

A major limitation of inhibitory chaperones is the optimization of drug dosing to balance their chaperoning and inhibitory capacities, as discussed in the previous section. Non-inhibitory chaperones promote the folding of mutant GCase in the ER and translocate it to lysosomes by binding to a site that differs from the active site, thus directly inducing the residual activity of mutant GCase (Baden et al., 2019), making non-inhibitory chaperones promising candidate pharmacological targets. A novel non-inhibitory GCase chaperone, NCGC607, which was identified by high-throughput screening, was demonstrated to restore GCase activity and reduce glycolipid storage in dopaminergic neurons of patients with GD, highlighting the potential of NCGC607 treatment for GD (Aflaki et al., 2016). Additionally, NCGC607 was capable of reducing α-synuclein levels in dopaminergic neurons of patients with parkinsonism, indicating its potential as a therapy for PD (Aflaki et al., 2016). Another non-inhibitory small-molecule chaperone, NCGC758, was reported to enhance GCase activity and reduce the level of GlcCer, ultimately enhancing the clearance of α-synuclein in iPSC-derived dopaminergic neurons of PD and GD patients (Mazzulli et al., 2016). Overall, pharmacological small-molecule chaperones have exhibited some advantages over standard therapy, including ERT and SRT. Small-molecule chaperones not only exhibit the ability to cross the BBB but can also be orally administered and are inexpensive to manufacture.

## Summary and Future Perspectives

*GBA* mutations are the most significant and common genetic risk factors associated with PD and are especially common in the Ashkenazi Jewish population. The mechanism by which *GBA* mutations result in an increased risk of developing PD remains unclear. Several mechanisms have been suggested to contribute to the pathogenesis of *GBA*-associated PD, including enhanced α-synuclein aggregation, autophagy-lysosomal dysfunction, altered lipid homeostasis and mitochondrial dysfunction. Both gain-of-function and loss-of-function explanations have been proposed to describe the pathogenesis of *GBA*-associated PD. The gain-of-function theory suggests that mutant GCase increases α-synuclein aggregation, leading to neurodegeneration. Alternatively, enhanced α-synuclein aggregation results in lysosomal dysfunction or impairment of autophagy, subsequently contributing to the development of parkinsonism. The loss-of-function theory posits that parkinsonism arises as a result of GCase deficiency, which influences lysosomal degradation function, negatively affecting α-synuclein turnover and substrate degradation. However, each of the models and potential pathways attributed to these theories has limitations. First, neither theory adequately explains the reason that only a fraction of GD patients and *GBA* carriers develop PD. Second, some patients with PD express *GBA*-null alleles; for example, those with c.84dupG also develop PD, in conflict with the gain-of-function theory. *GBA* carriers with null alleles may exhibit a higher risk of developing PD (Gan-Or et al., 2015). The current mechanisms can only be supported when *GBA* is mutated and has an enhanced effect on neurodegeneration but is not the initiator of pathogenesis. Other factors may contribute and may help determine whether mutant *GBA* can disrupt the cellular homeostatic system and subsequent α-synuclein pathology and PD, to an extent. Alternatively, *GBA* may act as a ‘second hit’ in some carriers and patients who are genetically predisposed to developing PD in the presence of other factors, such as aging and environmental factors.

The activity of GCase is diminished in the substantia nigra of brains with *GBA* mutations; however, it is also decreased in sporadic PD brains (Gegg et al., 2012). These findings suggest that therapies modulating GCase may not only be applied for the treatment of *GBA*-associated PD but may also provide a novel therapeutic target for sporadic PD. Importantly, AAV-mediated GCase expression reduced the accumulation of substrate and α-synuclein in both early and late symptomatic GD mice and was effective in reversing cognitive impairment when added before or after the protein aggregate (Sardi et al., 2013). These data demonstrate that modulating GCase activity may be beneficial before or after the diagnosis of PD to prevent the onset of PD or impede the progression of some aspects of GD-associated parkinsonism and PD. In general, the understanding of the function of GCase and the link between *GBA* mutations, α-synuclein accumulation and PD have made a significant contribution to the thinking on the pathogenesis of PD, and modulation of GCase may be beneficial to all PD patients, GD patients and those with other synucleinopathies. Although many studies have focused on the association between *GBA* mutations and PD, the GCase story may remain incomplete. Further exploration into the role of this enzyme in PD pathogenesis, manipulation of the GCase pathway, and potential genetic modifications may enable us to better understand the pathogenetic factors in the etiology of PD.

## Author Contributions

WZ designed and prepared the manuscript. DF supervised the manuscript. Both authors contributed to the article and approved the submitted version.

## Conflict of Interest

The authors declare that the research was conducted in the absence of any commercial or financial relationships that could be construed as a potential conflict of interest.

## Publisher’s Note

All claims expressed in this article are solely those of the authors and do not necessarily represent those of their affiliated organizations, or those of the publisher, the editors and the reviewers. Any product that may be evaluated in this article, or claim that may be made by its manufacturer, is not guaranteed or endorsed by the publisher.
